# Machine learning–assisted optimization of a terahertz photonic metamaterial absorber for blood cancer detection

**DOI:** 10.1371/journal.pone.0340492

**Published:** 2026-02-05

**Authors:** Asad Miah, Sams Al Zafir, Joyonta Das, Jonayed Al-Faruk, Shadi Ihtiaj Zim, Rafi Ahmad, Md. Rifat Hossen, S.M. Anowarul Haque, Abdul Wahed

**Affiliations:** 1 Department of Electrical and Electronic Engineering, Mymensingh Engineering College, Mymensingh, Bangladesh; 2 Department of Computer Science and Engineering, Mymensingh Engineering College, Mymensingh, Bangladesh; 3 Department of Information and Communication Engineering, Pabna University of Science and Technology, Pabna, Bangladesh; Parul University Parul Institute of Technology, INDIA

## Abstract

Blood cancer originates in the bone marrow and disrupts the body’s normal hematopoietic processes. The rapid progression of this disease highlights the need for accurate and sensitive detection to improve treatment outcomes. Here, we present a novel, compact, multi-resonant terahertz photonic metamaterial absorber for blood cancer detection. The compact structure, with dimensions of 0.41$\lambda_{0}$ × 0.41$\lambda_{0}$ × 0.029$\lambda_{0}$, integrates multiple circular resonators with a rectangular patch, supporting strongly confined resonances that achieve near-unity absorption of 97.6%, 99.9%, and 99.6% at 3.48 THz, 4.95 THz, and 6.01 THz, respectively. The sensing performance was evaluated by introducing an analyte layer representing normal and cancerous blood cells, resulting in remarkable sensitivities of 1.28 THz/RIU, 3.00 THz/RIU, and 2.14 THz/RIU at the three resonance frequencies. The corresponding quality factor (Q-factor) values are 15.03, 20.21, and 24.5, and the figure of merit (FOM) values are 5.54, 12.24, and 8.73 for the three resonance peaks, supporting its reliability in sensing. Moreover, the electric field, magnetic field, and surface current distributions were analysed, and an equivalent circuit model was also developed and validated against the simulated results. Several machine learning models were also employed for design prediction, with Gradient Boosting demonstrating excellent performance and enabling up to a 60% reduction in optimization time. The combination of a multi-band, high-absorption design and ML-assisted approach provides a robust, ultrathin, and high-sensitivity platform, offering a promising route toward next-generation terahertz biophotonic sensors for accurate and sensitive blood cancer detection.

## Introduction

Cancer is one of the most devastating diseases humans have ever faced, and it is estimated that by 2030, over 17 million people will have died as a result [1]. Among the several types of cancer, blood cancer is notable for its invasive character. It begins in the bone marrow and disturbs the body’s normal haematopoietic function by causing the excessive proliferation of aberrant blood cells. Proliferation disrupts the creation and function of healthy blood cells, affecting the immune system and physiological equilibrium [2]. Every year, the incidence of blood cancer rises due to a variety of factors, including genetics and the environment. Though techniques such as dose-intensification are beneficial for younger patients and produce promising results, they do not work for older individuals [3]. Other techniques, such as the electrochemical method, lack specificity for cancer cells and have limited ability to identify intracellular protein markers [4]. Furthermore, techniques such as fluorescence imaging and cytometry increase both cost and operating time [5]. Nowadays, biosensors are increasingly appealing due to their versatility in sensing. A good sensor should be susceptible to even minor changes in the analytic layer and linear over a frequency range. It should also be capable of doing real-time analysis and providing immediate feedback, which is critical for making fast decisions in medical contexts [6]. An optical biosensor is another sensor that can be perfect for bio-sensing due to its high sensitivity, small size, and low cost. Optical sensors have several types, including photonic crystals, metamaterials, optical fibers, etc [7–9].

Metamaterials have recently received more interest due to their unusual electrical and magnetic properties. Because its qualities are not present in nature and depend only on structure and organisation, high absorption can be produced [10–12]. These qualities make it a versatile element that can be used in a variety of applications, including solar energy harvesting, cloaking devices, imaging, antenna-based sensors, early-stage cancer detection, and RI sensing [13–19]. A seven-peak MMA is presented [20] for sensing applications in the biological field. The most excellent absorption value obtained in the investigation was 98.7, while the sensitivity calculated was 4.72 THz/RIU. Furthermore, they suggested a machine learning implementation to optimise the design structure and discovered that using the ERT model can save up to 60% simulation time. [21] describes a metamaterials-based sensor for detecting blood cancer. This sensor operates within the 0.6 to 1.2 THz range, exhibiting absorption rates exceeding 95% across the entire band. The sensitivity was calculated for three bands and found to be 0.435, 0.7, and 1.29, with the magnetic wire interference approach also being used to detect normal and blood cancer cells. A sensor based on MMA with four absorption peaks has been proposed for microbial sensing [22]. This investigation operates between 0 and 3.8 THz, calculating sensitivity values of 0.103 and 0.095 THz/RIU. A THz metamaterial sensor for detecting blood cancer is demonstrated [23]. This sensor has a substantial absorption value, and it calculates sensitivity for three peaks. Though it is difficult to obtain a decent sensitivity rating when evaluating multimode sensing, this sensor works well, detecting 0.93, 1.63, and 2.54 THz/RIU for its three peaks. This study also discusses the imaging technology used to discern between normal and malignant blood cells. [24] demonstrates a multi-band metamaterial sensor in the THz area. This study discovered good absorption values for various frequency peaks and then used machine learning for design optimisation. They tested many models, including KNN, ExtraTree, and CatBoost, and discovered that the KNN model produced the best results. A polarization-insensitive MMA with more than 99% absorption at triple band frequencies is presented [25] for biological applications. This sensor offers a good Q-factor value, with the most incredible sensitivity value of 1.08 THz/RIU. They test their sensor for cervical cancer cells and use the MWI approach to discern between normal and cancerous situations. An adjustable MMA in the THz range is described [26] for biological sensing. This study identified two frequency peaks with absorption values greater than 98%. The most excellent sensitivity was 1.15 THz/RIU, with q factor and fom values of 1.55 and 8.92. As detecting subtle dielectric variations is a major challenge, an MTM-based sensor was designed and fabricated in [12] for liquid chemical detection in the 8–12 GHz range. The authors tested various samples and observed clear frequency shifts corresponding to their dielectric properties. Furthermore, a triple-band metamaterial absorber with near-perfect absorption was proposed in [27] for terahertz applications. This structure is polarization-insensitive and demonstrated absorption values above 99% at all resonance peaks. The reported sensing performance includes a depth sensitivity of 2.76 THz/*μ*m and a refractive-index sensitivity of 1.55 THz/RIU. In [28], a quadrilateral half-circle resonator metasurface was introduced for K-band microwave applications, exhibiting strong absorption characteristics along with promising sensitivity, figure of merit (FOM), and a Q-factor of 55. For early-stage breast cancer detection, a compact Vivaldi antenna was proposed in [29], featuring a butterfly-shaped radiator. By exploiting dielectric contrast and microwave imaging, the antenna was able to distinguish between malignant and healthy tissues with improved accuracy. A metamaterial-inspired sensor was designed and fabricated in [30] to detect branded and unbranded diesel samples. During sensitivity testing, a variation of -3.2 dB was observed between the two types of diesel, demonstrating the sensor’s potential for fluid detection in chemical and medical industries. For early-stage breast cancer detection, which can yield a remarkable 99% survival rate, a microwave imaging technique was introduced in [31]. The study proposed a low-cost antenna array with an AMC structure, measuring 37.2 × 37.2 mm^2^. The prototype was fabricated, and the experimental results closely matched the simulations. Using the microwave imaging technique, the system successfully distinguished between normal and cancerous cells. Additionally, a symmetrical four-capacitance-loaded CCSRR triple-band metamaterial structure was presented in [32] for imaging, stealth, and thermal energy harvesting applications. The design exhibited polarization and incidence-angle insensitivity and achieved three nearly perfect absorption peaks.

We observed that several studies in the biomedical sensing field primarily rely on single-channel sensitivity, which limits detection accuracy. In contrast, our proposed multi-channel approach significantly improves sensing efficiency and reduces errors by operating across multiple frequency ranges and providing independent detection channels [33]. In addition, previous research on blood cancer sensing often reported low to moderate sensitivity values, and in many cases, the absorption performance degraded notably when tested with blood cell samples. Moreover, most of these studies did not explore machine learning-assisted optimization, which could accelerate the design process by reducing the extensive simulation time required to identify optimal structural parameters. Therefore, the objective of this study is to design a compact multi-channel terahertz metamaterial absorber–based sensor integrated with machine learning to achieve high-accuracy, high-sensitivity blood cancer detection with reduced simulation time.

To address these challenges, we propose a novel, compact terahertz metamaterial (MTM) sensor integrated with machine learning for blood cancer detection. The structure is extremely thin in width, height, and thickness, yet achieves high, stable absorption values that remain consistent even after introducing the analyte layer. The proposed design demonstrates superior multi-channel sensitivity compared with previously reported studies, thereby overcoming the limitations of low performance and absorption degradation. To further validate the design, an equivalent circuit model was developed in ADS and compared with full-wave simulations, showing excellent agreement. The electromagnetic response was analyzed through electric field, magnetic field, and surface current distributions, and the effect of varying incident angles was also investigated. Furthermore, several machine learning models were implemented for parameter prediction, with Gradient Boosting showing the best performance, reducing simulation time by up to 60% and enabling rapid design optimization. Overall, the proposed MTM sensor exhibits outstanding absorption, high multi-channel sensitivity, and robustness, making it a highly promising candidate for reliable blood cancer detection.

## Design and analysis

**Fig 1:**
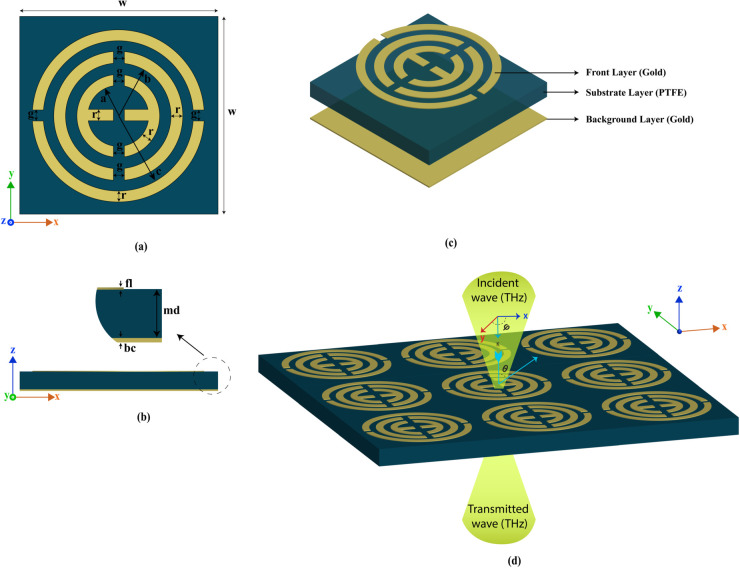
Proposed structure simulation setup.

The proposed structure consists of three separate layers, where one is a background layer made with gold, followed by the substrate layer and the top layer. In the back and front layer gold is used which posses several physical properties such as high electric conductivity (*σ* = 4.561e7 Sm^−1^), thermal conductivity of (*k* = 314 W/K/m), heat capacity (Cs) = 0.13 J/K/Kg, density = 19,320 kg/m^3^, Poisson’s ratio = 0.42, Young’s modulus = 78 GPa. Teflon, or PTFE, is used as a substrate material packed between two gold layers, which has an electrical conductivity of 2.1. The size of the background gold layer is 0.2 *μ*m, which helps prevent reflection and achieve the maximum absorption value for this structure. The front gold structure is also fragile, with a thickness of just 0.1 *μ*m, and the substrate thickness is 2.2 *μ*m. The front structure is designed by combining and arranging several circular structures. The entire layout is designed and simulated in CST Microwave Studio, which solves Maxwell’s equations using the Finite Integration Technique (FIT) in the time domain and the Finite Element Method (FEM) in the frequency domain, allowing accurate analysis of complex electromagnetic interactions, field distributions, and resonance characteristics within the metamaterial structure [34]. For the first circular c, this can be designed using the cylinder option, which offers several design flexibility options. For instance, for the design of the first circle, a circle is first inserted, followed by another circle with a lower radius. The two circles are then cut to create the outside structure. However, in a cylinder shape, two options are available: outer radius and inner radius. Another option is segments, where a value of 0 means the structure should be circular. This option helps us design the three circles, and then we added a rectangle shape with a length of 14 *μ*m and a resonator thickness of 2 *μ*m inside the smallest circle. After that, the top layer is cut into left and right sides with a two *μ*m size, and the other two are cut into top and bottom sides, which gives an epsilon shape in the smallest circle. The overall size of this structure is very compact, measuring 35.5 × 35.5 *μ*m^2^. The details parameter for this structure is listed in Table 1. These parameters were initially chosen based on an analysis of previously published high-performance designs and related literature. We then conducted extensive parametric studies to evaluate the influence of each parameter and identify the optimal configuration. The final set of parameters, which provided the best overall performance, was selected for this study. Fig 1(a) illustrates the entire structure, indicating the parameter. Furthermore, Figs 1(b) and (c) present the side view and 3D view of the structure. Finally, Fig 1(d) illustrates the incident wave in the structure with the incident and polarisation angle for the layout, which is 0 degrees. For the simulation, we use a different option with the Floquet port boundary condition. For the x- and y-axes, unit cell boundary conditions were applied, and an open additional space was used along the z-axis. The structure was excited with a plane wave incident along the z-direction to analyze its resonant response.

**Table 1:** Different parameter for this structure.

Parameters	Sizes (μm)	Parameters	Sizes (μm)
a	15.5-13.5	w	35.5
b	11.5-9.5	g = r	2
c	7.5-5.5	fl	0.1
md	2.2	bg	0.2

**Fig 2:**
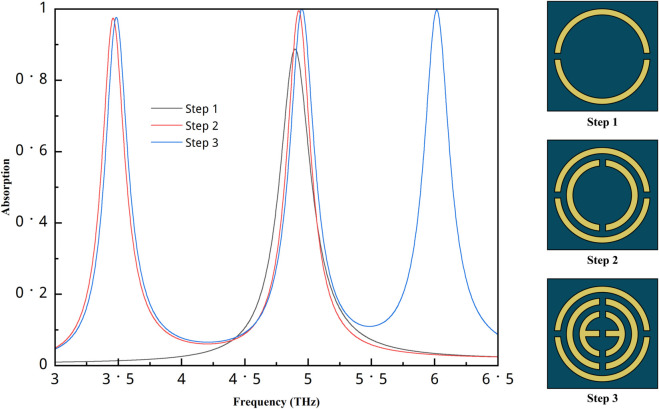
Step-by step design procedure and absorption result.

The step-by-step design procedure with the simulation result is shown in Fig 2. In the first step, only one absorption peak with more than 80% is found for the outer circle. After that, when another circle is added, another peak appears, and both peaks show high absorption values with good performance. Finally, another circle is added inside these circles, and a rectangle is added, forming the appearance of the epsilon symbol. With this configuration, we found three absorption peaks, and all of them show high values. Although the second structure shows two strong absorption peaks, our objective was to achieve a triple-band response. Introducing the third resonance enables sensing at multiple frequency bands, which helps reduce measurement error and improves overall sensing accuracy. Therefore, even though the absorption levels between Step 2 and Step 3 do not differ dramatically, the addition of the third peak provides a significant functional advantage for multi-band sensing applications.

**Fig 3:**
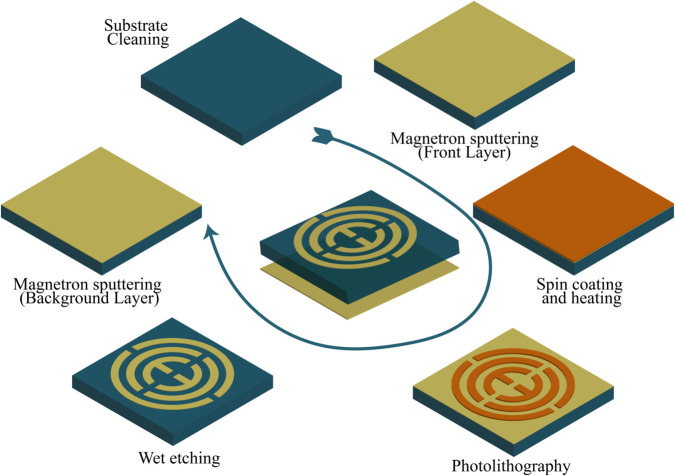
Possible fabrication process with steps.

The structure can be fabricated using the photolithography and wet etching techniques [35]. The possible fabrication method using this method is illustrated in Fig 3. Here, the substrate is first cleaned, and then gold is deposited on top of it using the magnetron sputtering method. After that, it undergoes a spin coating and heating stage, allowing us to build the structure further using photolithography methods. Wet etching is applied, which completes the front structure. For the backside, gold is again deposited using the magnetron sputtering method, which makes the overall structure. Additionally, this structure can be easily fabricated using Inkjet printing methods, which are more accurate and cost-effective [36]. As this is a thin-film structure, its fabrication presents several challenges. Maintaining the integrity of micro- and nanoscale dimensions requires strict control over process tolerances. Material-related issues may arise, including dielectric losses during processing, difficulties in creating complex masks, and potential material incompatibilities. Although multilayer structures with intricate geometries can be challenging to fabricate, our design is relatively simple, which helps mitigate these concerns. Environmental factors such as humidity and temperature may also influence the fabrication outcome [37–40]. Therefore, a sophisticated and well-controlled fabrication process, as outlined in the mentioned methods, is necessary to ensure reliable device realization.

### Ethics statement

This study is entirely simulation-based, and all biological parameters were obtained from previously published research articles. No human participants or animal subjects were involved in this work.

## Result analysis

### Absorption analysis

The absorption is an essential aspect of metamaterial research because metamaterial absorbers provide maximum interaction with incident light. To determine the absorption for this structure, use the following equation [37]:

A(ω)=1−R(ω)−T(ω) (1)

Here, A(ω) defines absorption, which is clearly dependent on Reflection R(ω) and Transmission T(ω). R(ω) can be substituted with the reflection coefficient $|S_{11}{|}^{2}$, and T(ω) with $|S_{21}{|}^{2}$. Now, Eq 1 can be written as

$$A(\omega )=1-|S_{11}{|}^{2}-|S_{21}{|}^{2}$$ (2)

When employing the back gold layer, there is no transmission in this structure, hence $T(\omega )=|S_{21}{|}^{2})$, and Eq 2 becomes,

$$A(\omega )=1-R(\omega )=1-|S_{11}{|}^{2}$$ (3)

Eq 3 is used to determine the absorption value for this structure.

The phenomenon of obtaining maximum absorption value can be described using the space charge equation [41].

$$A(\omega )=1-R(\omega )=\frac{Z_{w}-n_{0}}{Z_{w}+n_{0}}=\frac{z(w)-1}{z(w)+1}$$ (4)

**Fig 4:**
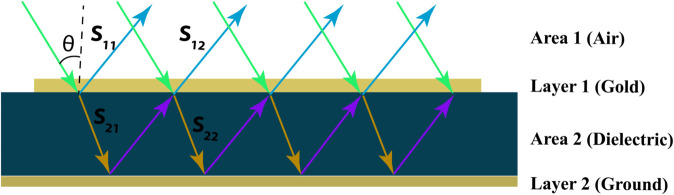
Schematic of interference model for this proposed MMA.

$Z_{(}\omega )$ denotes the wave impedance, and the normalised wave impedance, z(ω), is calculated using the S-parameters. *n*_0_ represents the impedance of free space. Maximum absorption occurs when the fundamental part of the normalised impedance is close to one and the imaginary part is close to zero, as given in Eq 2. This criterion indicates that the absorber’s impedance should match that of open space. The reflection coefficient is defined as $R={\left|\frac{Z-Z_{0}}{Z+Z_{0}}\right|}^{2}$ [42]. $Z=\sqrt{\mu /\epsilon }$ represents the medium’s impedance, while $Z_{0}=\sqrt{\mu_{0}/\epsilon_{0}}$ denotes the impedance of the surrounding environment. When the two impedances are equal, reflection becomes zero (R=0). In this case, the absorption reaches its maximum, as described by the equation A(ω)=1. Because the background metallic layer precludes transmission, the reflection coefficient is the only factor determining perfect absorption. The interference theory is frequently used to investigate this reflection behaviour further. In this theory, the top metallic resonator layer (referred to as Layer 1) serves as the reference point for position 0. The open air is designated Area 1, while the substrate is handled as Area 2. The schematic view of the interference model is shown in Fig 4, clearly illustrating the designated areas, layers, and the corresponding S-parameters.

$r_{11}=|r_{11}|e^{j\theta_{11}}$ represents the reflection coefficient from Area 1 back into Area 1 via Layer 1, whereas $t_{21}=|t_{21}|e^{j\theta_{21}}$ represents the transmission coefficient from Area 1 into Area 2 via Layer 1. Similarly, $t_{12}=|t_{12}|e^{j\theta_{12}}$ represents the transmission from Area 2 back to Area 1, whereas $r_{22}=|r_{22}|e^{j\theta_{22}}$ represents the reflection within Area 2. The overall effective reflection coefficient for Layer 1 is stated as follows [43]:

$$\sum r_{11}=|r_{11}|e^{j\theta_{11}}+\frac{|t_{12}{|}^{2}e^{j(\theta_{12}+\theta_{21}-2\beta -\pi )}}{1-|r_{22}|e^{j(\theta_{22}+\theta_{21}-2\beta -\pi )}}$$ (5)

*B* = *kd* denotes the propagation phase, where *d* is the wave’s distance from Layer 1 to the ground plane and *k* is the wavenumber. The equation demonstrates that the total reflection is composed of two contributing components; when these components have similar magnitudes but opposing phases, they interfere destructively and cancel each other out. This causes zero reflection, resulting in maximum absorption.

**Fig 5:**
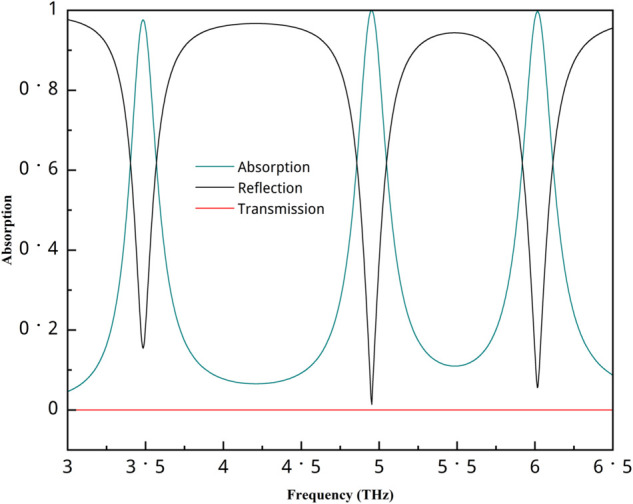
Absorption, reflection, and transmission for this structure’s.

Fig 5 depicts the absorption, reflection, and transmission coefficients for this configuration. The transmission is zero due to the rear gold layer. The absorption values for all peaks are incredibly high. The absorption values are 97.6%, 99.9%, and 99.6% at frequencies of 3.48 THz, 4.95 THz, and 6.01 THz, respectively. We also calculated the Full Width at Half Maximum (FWHM), which is defined as the frequency range between the lower and upper points at 50% of the peak value. In this study, we performed the analysis using OriginPro software, which provides excellent capabilities for precise data processing and visualization. The FWHM values for these frequencies are 0.2318, 0.24501, and 0.24529. The quality factor (Q-factor) is an essential parameter for determining the performance of a resonant frequency mode, as a higher Q-factor indicates stronger resonance and consequently improves sensitivity and efficiency, making the device more suitable for high-performance sensing applications. The Q-factor is calculated using the equation $Q=f_{c}/FWHM$ [20], where *f*_*c*_ represents the resonance frequency. The calculated Q-factor values are 15.03, 20.21, and 24.5 for the three frequency peaks, respectively.

### Equivalent circuit analysis

**Fig 6:**
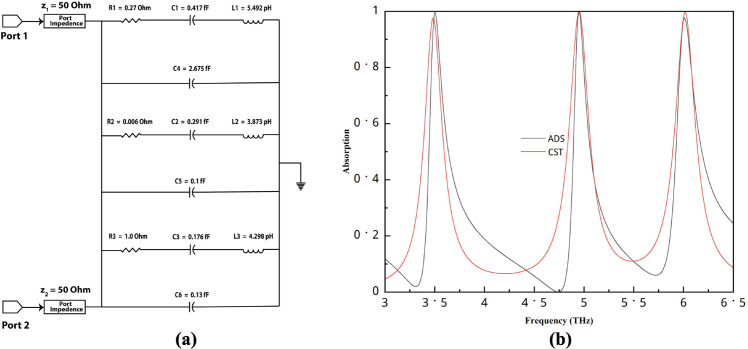
(a) Equivalent circuit using ADS, (b) Comparison of ADS and CST results.

To validate the proposed structure, an equivalent circuit was created using ADS software, as shown in Fig 6(a). In this model, the split gap and the gap between the two stripes are represented as capacitors, while the microstrip lines are modelled as inductors. The circuit was tuned in ADS, and the resulting response closely matches the full-wave simulation results, as compared in Fig 6(b). The resonance peaks occur at almost the same positions, particularly for the second and third bands. Some discrepancies are observed in the lower part of the graph, which can be attributed to the fact that CST simulation results represent a full-wave, static solution, whereas the ADS circuit requires parameter tuning to approximate the electromagnetic response.

### parametric study analysis

**Fig 7:**
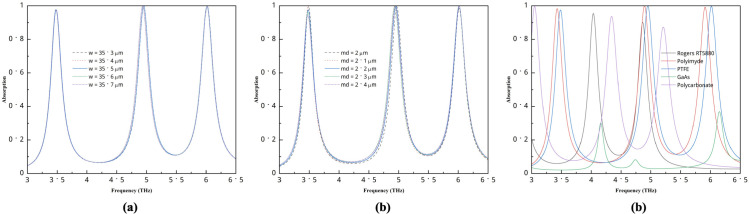
Parametric studies of structure (a) different width and height, (b) substrate thickness, (c) different materials.

Several parametric study was conducted to find the best structure for this study. For different widths and heights ranging from 35.3 *μ*m to 35.7 *μ*m, the value is almost identical for the first and third peaks shown in Fig 7. In contrast, the value of the second peak remains unchanged but is slightly shifted. For this structure, we can use any of the widths; however, for optimal overall performance, a width of 35.5 *μ*m and height of 35.5 *μ*m are used. The two *μ*m and 2.1 *μ*m substrate thicknesses increase the first band absorption value, but they reduce the second and third band absorbance values. Here, 2.2 *μ*m values yield the perfect result, and then the first one reduces slightly, so we use it for this layout. Finally, we study several materials used as a substrate. Here, Rogers RT5880 gives two peaks with good and average absorption values. Polycarbonate exhibits three frequency peaks, with one having a high absorption value and the other two being average. GaAs performs the worst, offering two small peaks. Polyimide and PTFE perform almost similarly, and their first absorption peak is better than PTFE, but other peaks show a slightly lower performance; thus, PTFE is used as a substrate in this structure.

### Angle stability

**Fig 8:**
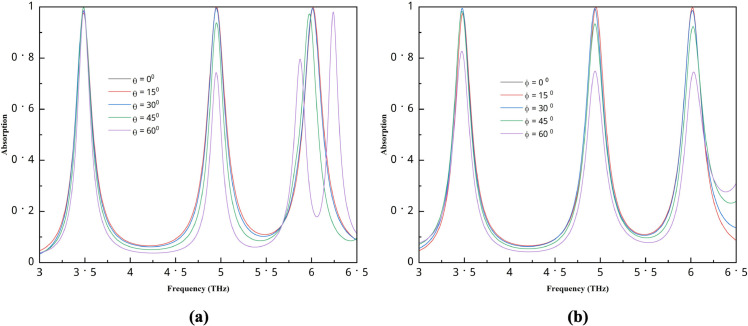
Absorption performance for different (a) incident angle, (b) polarization angle.

Fig 8(a) shows the absorption performance for different incident angles. The performance is stable between 0 and 15 degrees. At 30^0^, it still shows good performance and is almost in the same position as 0 and 15 degrees. Then, the 45-degree angle performs slightly lower, and only the first peak performs well for all angles. In contrast, for 60^0^, the second and third peaks do not perform well, with an additional peak observed. The same phenomenon occurs for the polarization angle shown in Fig 8(b), where 0 and 15 degrees are almost identical, and at 30^0^, the absorber performs well. Then, the performance slightly drops, as the second and third peaks for 45^0^ show a somewhat lower absorption value, and for 60^0^, all the peaks perform at an average level. This structure can perform well between 0 and 30 degrees, after which its performance may decrease slightly. However, the first peak still performs very well at larger angles, so it may be used if needed. Nevertheless, a 30-degree angle is sufficient for our purposes, as we consider light to come in at 0 degrees.

### E-field, H-field and surface current distributions

**Fig 9:**
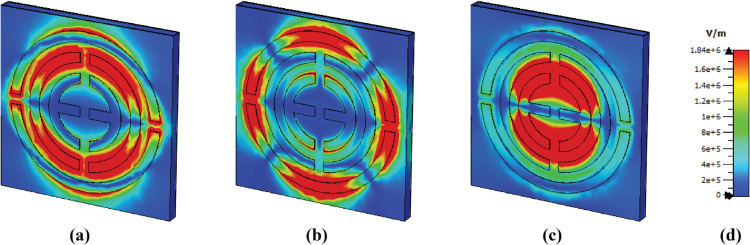
E-field distribution at (a) 3.48 THz, (b) 4.95 THz, and (c) 6.01 THz.

The e-field distribution for different frequencies is shown in Fig 9. Several areas exhibit a high concentration of the e-field. Thus, it becomes red; this occurs due to surface plasmon resonance (SPR) [44]. For the first band, the top and bottom sides of the middle circle show a high concentration of electric field due to Localized surface plasmon resonance (LSPR) [45]. For the frequency of 4.95, the outer circular shape exhibits several high field intensities on various sides. The top and bottom are due to LSPR, but the left and right are due to Cavity surface plasmon resonance [25, 46]. For the last frequency, the top and bottom sides of the two middle circular layers show high concentration, where both CSPR and LSPR are observed.

**Fig 10:**
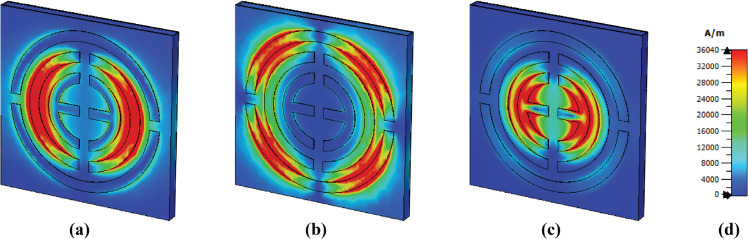
H-field distribution at (a) 3.48 THz, (b) 4.95 THz, and (c) 6.01 THz.

A higher magnetic intensity is observed on the left and right sides of the middle circular shape at a frequency of 3.48 THz, due to LSPR, presented in Fig 10. Then, several sides of the last circle show a higher concentration, while other shapes don’t show anything. For the last frequency, the epsilon shape has a higher magnetic field intensity due to LSPR.

**Fig 11:**
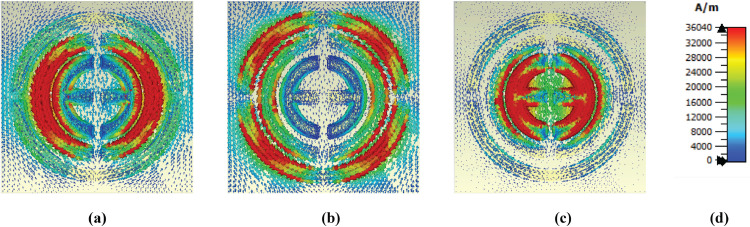
Surface current distribution at (a) 3.48 THz, (b) 4.95 THz, and (c) 6.01 THz.

High field intensity in the surface current is observed to be the same as that of the magnetic field shows in Fig 11. The middle circular shape shows a high concentration of surface current on its left and right sides. However, several antiparallel currents are observed on the side of these, which means a strong magnetic field is present in this area, as the presence of parallel and antiparallel currents means a strong magnetic field [47]. The outer structure exhibits high intensity at 4.95 THz in several of its areas, and there are also several antiparallel currents present, which means a potent magnetic field is also present here. Finally, at a frequency of 6.01 THz, a high surface current is observed in the epsilon shape and is slightly higher in the second layer.

## Sensing performance

**Fig 12:**
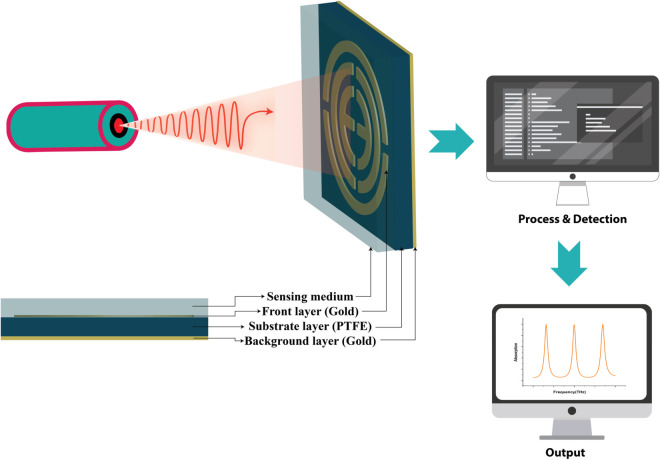
Sensor setup for this absorber.

The sensing performance of this absorber is also evaluated by adding an analytic layer at the top of the substrate. The top gold layer of this absorber is located inside the sensor, and the sensor size is 3 *μ*m. The sensor setup is depicted in Fig 12, where the terahertz source interacts with the structure, and the sensor sample of the cell is placed in a liquid form inside the sensor. When light hits the sensor, it is processed, and we can view different types of graphs for these samples.

### Blood cancer detection

**Fig 13:**
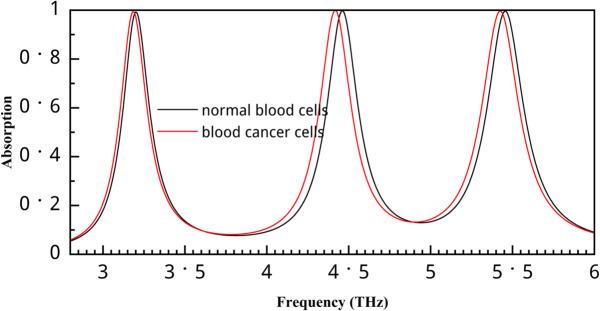
Absorption value for normal and cancer blood cells.

This study further evaluates the sensor’s performance in detecting blood cancer. As this study is simulation-based, it uses the refractive index of normal and cancer cells. The refractive index value of normal blood cells is 1.376, and that of cancer blood cells is 1.390 [21]. The absorption values for normal and cancerous blood cells are depicted in Fig 13. All three bands show significant absorption shifts for these conditions, and the second band performs the best. For normal conditions, the frequency values are 3.2 THz, 4.46 THz, and 5.456 THz, and for cancer conditions, they shift to 3.182 THz, 4.418 THz, and 5.426 THz. Sensitivity serves as a crucial performance metric for liquid sensors, reflecting their capability to detect and distinguish even minor variations in the permittivity of the target liquid. A highly sensitive sensor ensures accurate monitoring of subtle changes in the sample’s dielectric properties, which is essential for precise sensing applications [48]. The sensitivity is calculated using the following equation [17]:

$$S=\frac{\Delta f_{0}}{\Delta n}$$ (6)

Where $\Delta f_{0}$ is the difference between resonance frequencies and Δn is the difference in refractive index value. After calculation, we found the sensitivity of 1.28 THz/RIU for the first peak, 3 THz/RIU for the second peak, and 2.14 THz/RIU for the third peak.

Table 2 compares the sensitivity with previous research published in the biomedical field. This study compares the triple and double band structures, and the sensor performs very well in this regard. In [21], Hamza *et al*. Proposed a sensor for blood cancer detection; however, the sensitivity is low compared to our sensor. Their highest sensitivity is almost equal to our lowest sensitivity performance. After that, in [23], another triple-band sensor is proposed, and its sensitivity is relatively high; however, our sensors perform very well compared to it. Then, Bhati *et al*. [22] proposed a dual-band sensor for microorganism detection. Our senor performs very well compared to this and other previous published literature. Therefore, this could be an ideal choice for detecting blood cell cancer. The figure of merit (FOM), defined as the ratio of a sensor’s sensitivity to its full width at FWHM, is another important parameter for evaluating and comparing the sensing performance of different sensors. The Figure of Merit (FOM) is calculated using the standard definition [43]:

**Table 2:** Sensitivity comparison with previous published research.

Ref.	S (THz/RIU)	Publishing year	Publishing in	Applications
[21]	0.435, 0.7, 1.29	2025	Plos one	Blood cancer diagnostics
[22]	0.103, 0.095	2023	Scientific Reports	Microorganisms detection
[23]	0.93, 1.63, 2.54	2024	Plasmonics	Blood cancer diagnostics
[26]	0.571, 1.15	2025	Optics Express	Biomedical sensing
[47]	0.0515, 0.076	2023	IEEE Access	Skin Cancer Diagnostics
[49]	0.0968, 0.1182	2022	American chemical Society	Biomedical Application
[50]	0.13, 0.14	2024	Photon. Nanostruct. Fund. Appl.	sensing
[51]	1.4, 0.545, 0.229	2025	Optics and Laser Technology	Liquid sensing
[41]	1.5, 1 , 0.66	2025	Plos one	Biomedical Applications
Proposed	1.28, 3, 2.14	-	-	Blood cancer detection

$$FOM=\frac{S}{FWHM}$$ (7)

where *S* is the sensitivity and *FWHM* is the full width at half maximum of the resonance peak. Using the previously calculated sensitivity and corresponding *FWHM* values, the *FOM* values are 5.54, 12.24, and 8.73 for the three resonance peaks, respectively.

## Machine learning for absorption coefficient prediction

This section briefly examines the role of regression models in the simulation process, highlighting their potential to reduce the time and computational resources needed for efficient absorber design by approximately 40%, 50%, or even 60%. Regression analysis provides a systematic method for estimating dependent parameters based on independent variables. In this study, frequency is the independent variable in absorber design, while the absorption coefficient is the dependent variable. Designing and simulating complex structures usually requires significant time and resources; however, these challenges can be effectively addressed with machine learning (ML) regression techniques. These methods not only speed up the simulation process but also help in estimating missing parameter values. To address these issues, the proposed approach involves three stages of ML-based regression analysis.

Step 1: Simulate the absorber design by varying frequency with larger step sizes.

Step 2: Use the simulated data to train a machine learning regression model.

Step 3: Use the trained model to predict absorptivity at intermediate frequency values.

Step 4: Apply explainable AI (XAI) techniques, such as SHAP and LIME, to interpret the model’s predictions.

### ML model selection and performance evaluation

This study uses a dataset with 41,086 sensor configurations generated through CST Microwave Studio, based on systematic variations of eight key design parameters: split gap (g) (1.775–2.25 *μ*m), ring width (r) (1.775–2.25 *μ*m), substrate thickness (md) (2.05–2.5 *μ*m), and structure width and height a (32.5–37.5 *μ*m). The initial parameter values were selected based on the finalized design of the proposed structure, focusing on the key geometrical parameters that significantly influence its resonance behavior. After establishing the optimal baseline design, we defined parameter ranges centered around these values to ensure that the ML model explores realistic and physically meaningful variations. The ranges were kept uniform to maintain structural feasibility while remaining broad enough to capture meaningful performance differences. These selected geometrical parameters were then used as input features for the ML model, with the absorption magnitude serving as the target variable. After data preprocessing, which included noise and outlier removal, multiple validation schemes (80:20, 60:40, 50:50, and 40:60) were employed to assess the sensitivity and robustness of the machine learning models in predicting sensor performance. Various supervised regression algorithms were implemented, such as Gradient Boosting Regressor, Extra Trees Regressor, XGBoost (XGB), Random Forest Regressor (RFR), Decision Tree Regressor (DTR), and k-Nearest Neighbors (KNN) [52, 53]. Hyperparameter tuning was performed using BayesSearchCV, RandomizedSearchCV, and GridSearchCV to improve predictive accuracy [54, 55].

Table 3 displays the baseline performance without hyperparameter tuning. Among the models tested, RandomForest showed the highest accuracy (R^2^ ≈ 0.996), followed by DecisionTree and ExtraTrees. Ensemble methods consistently outperformed single estimators, while GradientBoosting achieved moderate results, and AdaBoost performed relatively weakly. In contrast, Table 4 presents the results of three hyperparameter optimisation methods, Grid Search, Randomized Search, and Bayesian Search, applied to different algorithms. The results show that Randomized Search with Gradient Boosting gave the best performance, reaching an R^2^ of 0.9991 and the lowest error metrics (MSE = 0.000029, RMSE = 0.0054, MAE = 0.0018). Similarly, both Grid Search and Bayesian Search identified RandomForest as the top model, with slightly lower but still reliable accuracy (R^2^ ≈ 0.996).

**Table 3:** Baseline performance of machine learning models before hyperparameter tuning.

Model	R^2^	Adj R^2^	MSE	RMSE	MAE
RandomForest	0.995875	0.995874	0.000138	0.011742	0.003732
DecisionTree	0.994353	0.994352	0.000189	0.013738	0.004081
ExtraTrees	0.993010	0.993008	0.000234	0.015285	0.004531
XGBoost	0.991034	0.991031	0.000300	0.017312	0.006723
KNN	0.980092	0.980086	0.000665	0.025796	0.007624
GradientBoosting	0.954282	0.954268	0.001528	0.039091	0.015128
AdaBoost	0.782540	0.782471	0.007269	0.085256	0.048981

**Table 4:** Comparison of hyperparameter tuning methods.

Tuning Method	Best Model	R^2^	Adj R^2^	MSE	RMSE	MAE
Grid Search	RandomForest	0.995943	0.995941	0.000136	0.011645	0.003709
Randomized Search	GradientBoosting	0.999125	0.999124	0.000029	0.005409	0.001761
Bayesian Search	RandomForest	0.995892	0.995891	0.000137	0.011717	0.003788

To further validate the predictive performance of the Gradient Boosting model, a 10-fold cross-validation was carried out using the k-fold method. As presented in Table 5, the model consistently attained coefficients of determination (R^2^) exceeding 0.999 across all folds, accompanied by remarkably low error values (RMSE ≈ 0.005; MAE ≈ 0.0018). Such outcomes demonstrate the model’s robustness and stability, while also underscoring its superior generalization ability. These findings highlight the reliability of Gradient Boosting as a powerful tool for precise and accurate prediction tasks.

**Table 5:** Cross-validation results for the Gradient Boosting model.

Fold	R^2^	Adj R^2^	MSE	RMSE	MAE
1	0.9991	0.9991	0.0000	0.0053	0.0017
2	0.9992	0.9992	0.0000	0.0051	0.0017
3	0.9992	0.9992	0.0000	0.0053	0.0018
4	0.9991	0.9991	0.0000	0.0057	0.0019
5	0.9990	0.9990	0.0000	0.0056	0.0018
6	0.9992	0.9992	0.0000	0.0052	0.0018
7	0.9990	0.9990	0.0000	0.0056	0.0018
8	0.9991	0.9991	0.0000	0.0055	0.0018
9	0.9991	0.9991	0.0000	0.0055	0.0018
10	0.9991	0.9990	0.0000	0.0058	0.0019

**Fig 14:**
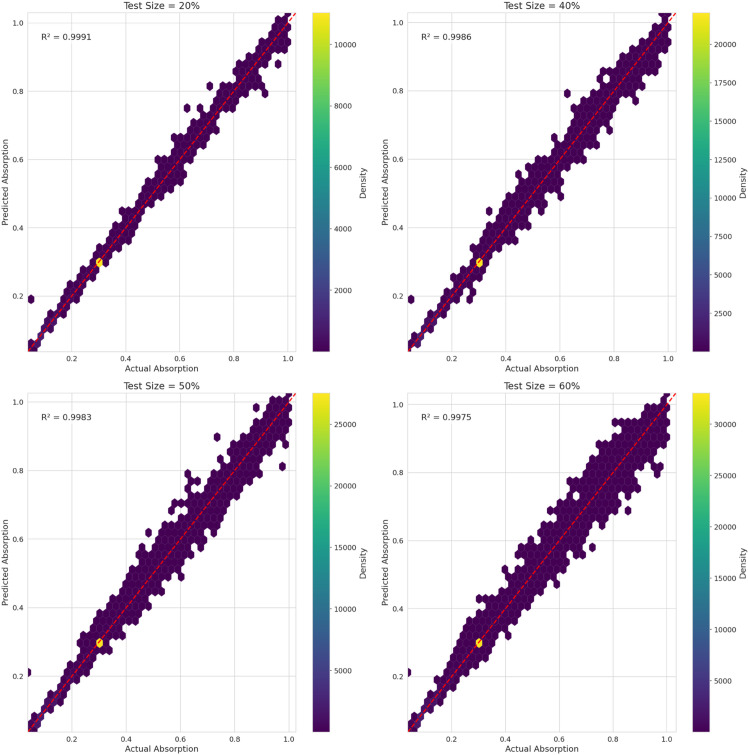
Scatter plot for gradient boosting.

**Fig 15:**
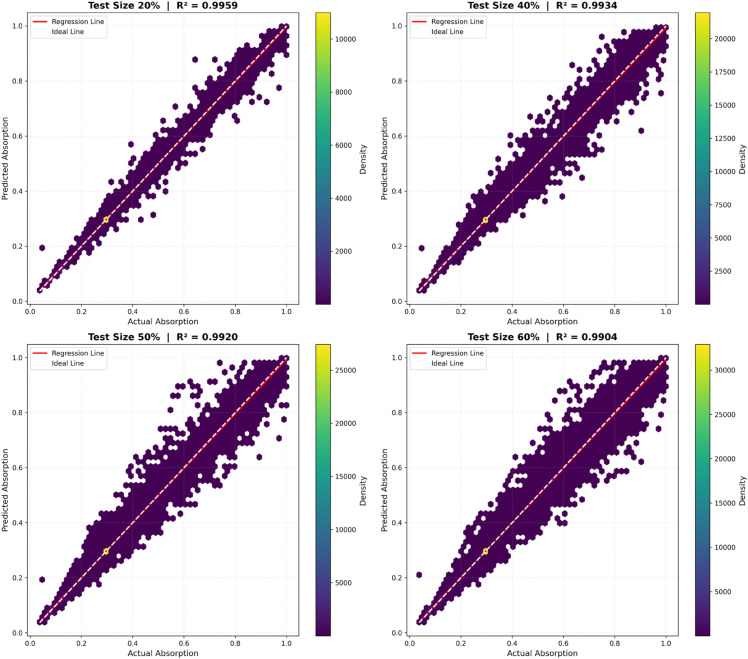
Scatter plot for random forest.

Figs 14 and 15 compare the performance of the two top-performing models, Gradient Boosting and Random Forest, under different test sizes (20%, 40%, 50%, and 60%), which correspond to varying degrees of experimental time reduction. The results show an outstanding performance at 20% (R^2^ = 0.9992 for Gradient Boosting; 0.996 for Random Forest) and an excellent performance at 40% (R^2^ = 0.9987 and 0.994, respectively). At 50%, the models continue to perform strongly (R^2^ = 0.9981 and 0.992), while at 60% they still achieve promising results (R^2^ = 0.9975 and 0.991). Overall, these findings confirm that both models, particularly Gradient Boosting, retain high predictive capability even at higher test proportions, demonstrating that our machine learning approach can effectively reduce experimental time by up to 60% without compromising accuracy.

**Fig 16:**
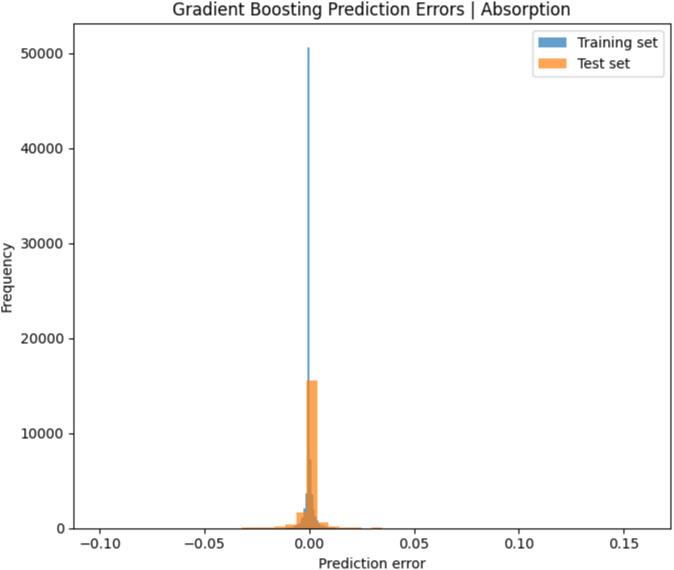
Prediction error of gradient boosting.

**Fig 17:**
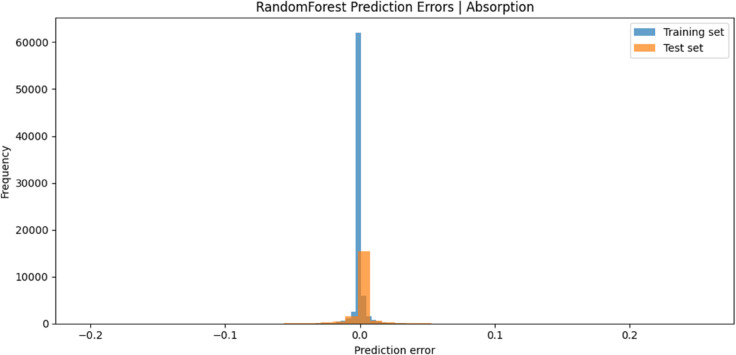
Prediction error of random forest.

Figs 16 and 17 present the prediction error distributions for the Random Forest and Gradient Boosting models, respectively. In both models, the error values for the training and test sets are predominantly concentrated around zero, reflecting minimal deviation between the predicted and actual absorption values. This narrow error distribution provides strong evidence of the high predictive accuracy and robust generalization ability of both approaches. Moreover, the Gradient Boosting model exhibits a comparatively more compact error spread than Random Forest, thereby reinforcing its position as the superior predictive method.

**Fig 18:**
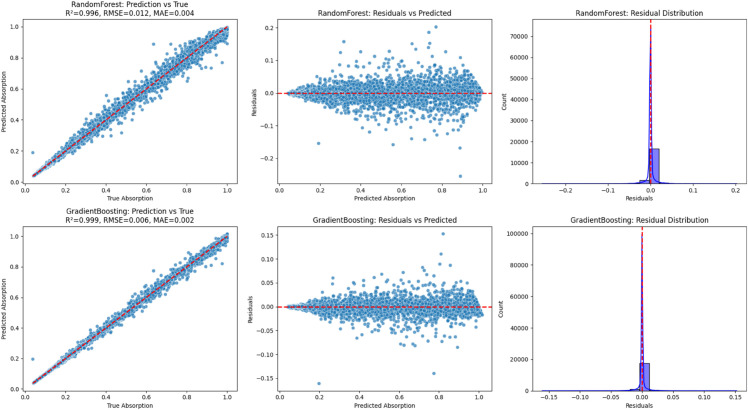
Predictive performance and residual behavior of the random forest and gradient boosting models.

Fig 18 compares the predictive performance and residual behaviour of the Random Forest and Gradient Boosting models. The prediction-versus-true plots (left column) indicate a strong alignment between predicted and actual absorption values, with Random Forest achieving R^2^ = 0.996, RMSE = 0.012, and MAE = 0.004. At the same time, Gradient Boosting demonstrates superior accuracy with RR^2^ = 0.999, RMSE = 0.006, and MAE = 0.002. The residual plots (middle column) further illustrate the distribution of errors. In both models, the residuals are tightly centred around zero, confirming minimal systematic bias. However, Gradient Boosting exhibits a narrower residual spread compared to Random Forest, indicating better stability across the prediction range. Finally, the residual distribution histograms (right column) reinforce these observations. Both models exhibit error distributions that are highly concentrated near zero, reflecting a strong generalisation capability. Yet, the sharper peak and tighter spread in Gradient Boosting highlight its advantage in delivering more precise and reliable predictions relative to Random Forest.

**Fig 19:**
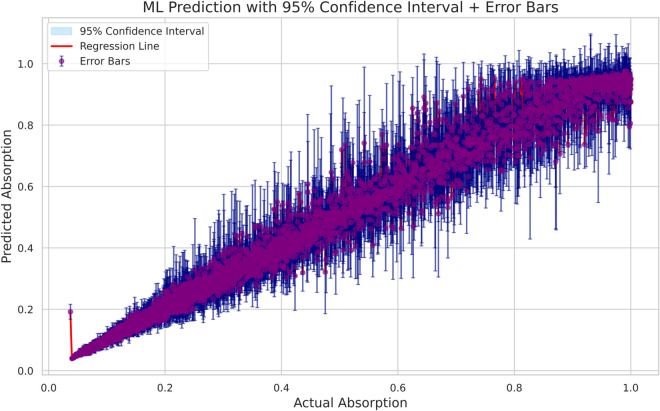
Gradient boosting prediction performance with 95% confidence intervals and error bars.

**Fig 20:**
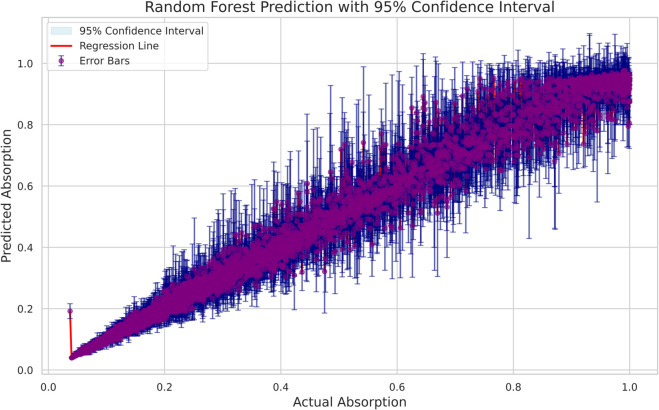
Random forest prediction performance with 95% confidence intervals and error bars.

The reliability of the machine-learning predictions was assessed through uncertainty estimation for both the Random Forest and Gradient Boosting models. For the Random Forest model, 95% confidence intervals (CI) were calculated using the ensemble mean and the variance across individual trees. The resulting plot (Fig 19 ), which includes a regression line, a shaded 95% CI band, and pointwise error bars, demonstrates that the majority of predictions fall within narrow intervals, indicating high predictive stability and low model uncertainty. For the Gradient Boosting model, bootstrap-based uncertainty estimation was applied, and the corresponding results (Fig 20 ) show a strong linear relationship between the predicted and actual absorption values. The confidence interval band and error bars similarly reveal that most predictions exhibit limited variability, with only minor dispersion at higher absorption levels. Overall, the uncertainty analyses confirm that both models provide accurate, stable, and reliable predictions across the full absorption range.

To ensure transparency and interpretability of the machine learning models, explainable artificial intelligence (XAI) techniques such as SHAP (SHapley Additive exPlanations) [56] and LIME (Local Interpretable Model-agnostic Explanations) [57] were employed. These methods are essential for understanding how individual features contribute to model predictions, thereby transforming black-box algorithms into interpretable frameworks suitable for scientific applications. SHAP provides both local and global interpretability by quantifying the positive or negative contribution of each feature to a prediction, while LIME approximates complex models locally with interpretable surrogates.

**Fig 21:**
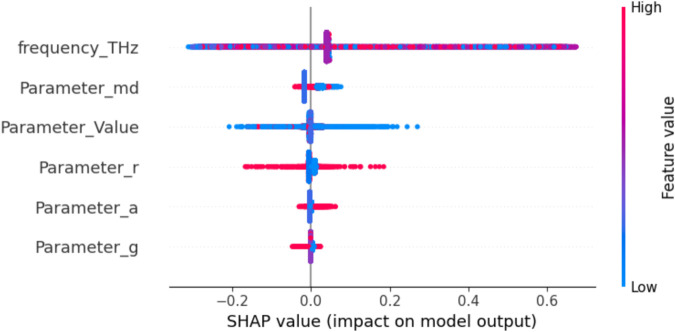
SHAP value impact on model output.

**Fig 22:**
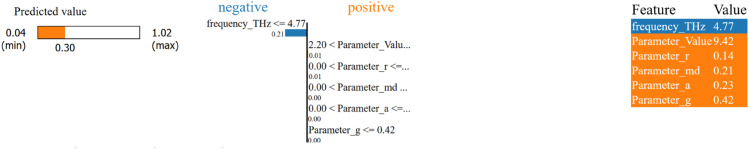
LIME explanation for the gradient boosting model.

Since Gradient Boosting was identified as the best-performing model, explainable artificial intelligence (XAI) techniques—specifically SHAP (SHapley Additive exPlanations) and LIME (Local Interpretable Model-agnostic Explanations)—were applied to enhance its interpretability. Figs 21 and 22 present the SHAP-based results. The local explanation (Fig 21) shows that the prediction value (0.30 within a range of 0.04–1.02) is strongly influenced by frequency_THz = 4.77, which exerts the most significant adverse effect, while Parameter_Value = 9.42, Parameter_r = 0.14, Parameter_md = 0.21, Parameter_a = 0.23, and Parameter_g = 0.42 contribute positively. The global summary (Fig 22) further confirms that frequency_THz is the most influential predictor, followed by Parameter_md and Parameter_Value, while the remaining parameters have more minor yet consistent impacts. By combining SHAP, which provides both global and local feature attributions, with LIME, which offers localized interpretability around individual predictions, the analysis highlights not only the dominant role of frequency in shaping predictions but also the complementary influence of secondary parameters. This dual interpretability approach strengthens the transparency, reliability, and trustworthiness of the Gradient Boosting model.

Table 6 presents a comparison of the proposed design with previously published works. The second and third absorption peaks exceed 99%, while the first peak also demonstrates strong performance. Compared with earlier studies, our absorber shows superior triple-band operation with consistently high absorption across all resonances. We have additionally compared the Q-factor and FOM with previous works. In many studies, these parameters were not reported, and in the few cases where they were provided, the values were generally lower than those obtained in our design. Only one study showed a higher Q-factor for a single peak, but it exhibited lower FOM, reduced absorption performance, and a significantly larger physical structure compared with our compact design. This comparison highlights the difficulty of achieving uniformly high absorption across multiple resonant peaks, a challenge clearly evident in the analyzed studies. The limitations observed in these works, such as inconsistent peak performance, larger structural dimensions, or lower Q-factor and FOM, further underline the advantage of our compact and well-optimized structure. Furthermore, none of the analyzed studies incorporated machine learning, whereas our approach integrates an ML-based absorption prediction model that significantly reduces simulation time while maintaining high accuracy. Overall, the compact and efficient structure, strong triple-band absorption, and improved Q-factor, FOM, and sensing performance demonstrate the clear advancement of our design, making it a promising platform for next-generation technology in blood cancer detection.

**Table 6:** Absorption comparison with previous works.

Ref.	Structure Size (μm)	Absorption	FOM	Q factor	ML Approach
[58]	100 × 100	95, 93, 96	0.67, 0.75, 24.48	0.15, 0.20, 0.049	No
[59]	120 × 120	96, 98, 95	6.93, 5, 4.3	29, 35, 46	No
[60]	-	97,98,99	-	-	No
[61]	80 × 80	99, 80, 95	-	-	No
[62]	35 × 35	90, 98.9	-	-	No
[63]	120 × 120	99.5, 86.4, 98.4	-	-	No
Proposed	35 × 35	97.6, 99.9, 99.6	5.54, 12.24, 8.73	15.03, 20.21, 24.5	Yes

Although the present study is entirely simulation-based, ADS is used to develop the equivalent circuit and validate the proposed design, while keeping fabrication feasibility in mind. We also discuss two well-known fabrication approaches that can be considered for experimental realization. After fabrication, the primary challenge will be testing with real biological samples. Before using actual samples, a dry-phantom setup that mimics the sensor response should be evaluated to verify performance and ensure safety. Additionally, measuring the complex permittivity of normal and cancerous blood and incorporating these values into simulations would help improve model accuracy. The investigation can then progress toward detecting real blood cancer, where testing human samples will require appropriate ethical approval. Intra-patient and inter-patient variability, as well as differences among blood-cancer subtypes, should also be evaluated to ensure reliable diagnostic performance. Finally, performance assessment should include statistical evaluation using sensitivity, specificity, and overall diagnostic accuracy.

## Conclusion

This study presents a square-shaped metamaterial structure for blood cancer detection. The ultrathin structure (35.5 × 35.5 *μ*m^2^) operates in the 3–6.5 THz frequency range, demonstrating triple-band absorption of 97.6%, 99.9%, and 99.6% at 3.48 THz, 4.95 THz, and 6.01 THz, respectively. The sensing performance was evaluated by introducing an analyte layer representing normal and cancerous blood conditions, achieving sensitivities of 1.28 THz/RIU, 3.00 THz/RIU, and 2.14 THz/RIU for the respective frequencies. The quality factor (Q-factor) and figure of merit (FOM) values were 15.03, 20.21, 24.5 and 5.54, 12.24, 8.73, respectively. The electromagnetic response, including electric field, magnetic field, and surface current distributions, was analysed, and an equivalent circuit was developed and validated against simulation results. Furthermore, several machine learning models were employed to predict optimal structural parameters. The Gradient Boosting model demonstrated excellent performance, achieving an R^2^ value of 0.9992 for 80% training data, and promising results for up to 40% testing data, indicating a potential 60% reduction in simulation time. Overall, the proposed metamaterial sensor exhibits high absorption and multi-band sensitivity, and the ML-assisted optimization provides a rapid and efficient design framework. Compared with previously reported single- and multi-band sensors, the proposed design demonstrates superior performance, highlighting its robustness, reliability, and potential for practical biomedical applications.

## Supporting information

S1 Data Relevant data for this study. (XLSX)
